# High level of soluble human leukocyte antigen (HLA)-G at beginning of pregnancy as predictor of risk of malaria during infancy

**DOI:** 10.1038/s41598-019-45688-w

**Published:** 2019-06-24

**Authors:** Tania C. d’Almeida, Ibrahim Sadissou, Mermoz Sagbohan, Jacqueline Milet, Euripide Avokpaho, Laure Gineau, Audrey Sabbagh, Kabirou Moutairou, Eduardo A. Donadi, Benoit Favier, Cédric Pennetier, Thierry Baldet, Nicolas Moiroux, Edgardo Carosella, Philippe Moreau, Nathalie Rouas-Freiss, Gilles Cottrell, David Courtin, André Garcia

**Affiliations:** 1 Université Pierre et Marie Curie, Paris VI, France; 2 MERIT, IRD, Université Paris Descartes, Paris, 75006 France; 3 IRD, UMR 261, Centre d’Étude et de Recherche sur le Paludisme Associé à la Grossesse et à l’Enfance (CERPAGE), Faculté des Sciences de la Santé, Cotonou, Benin; 4 Division of Clinical Immunology, School of Medicine of Ribeirão Preto, University of São Paulo, São Paulo, Brazil; 5 Université d’Abomey-Calavi, Cotonou, Benin; 6 Commissariat à l’Énergie Atomique et aux Énergies Alternatives, Direction de la Recherche Fondamentale, Institut de Biologie François Jacob, Service de Recherches en Hémato-Immunologie, Hôpital Saint-Louis, IUH, Paris, France; 7 Université Paris Diderot, Sorbonne Paris Cité, IUH, Hôpital Saint-Louis, UMR_E5, IUH, Paris, France; 8 UMR MIVEGEC (IRD-CNRS-UM), Montpellier, France; 9 Centre de Recherche Entomologiques de Cotonou (CREC), Cotonou, Benin

**Keywords:** Risk factors, Immunology

## Abstract

Placental malaria has been associated with an immune tolerance phenomenon and a higher susceptibility to malaria infection during infancy. HLA-G is involved in fetal maternal immune tolerance by inhibiting maternal immunity. During infections HLA-G can be involved in immune escape of pathogens by creating a tolerogenic environment. Recent studies have shown an association between the risk of malaria and HLA-G at both genetic and protein levels. Moreover, women with placental malaria have a higher probability of giving birth to children exhibiting high sHLA-G, independently of their own level during pregnancy. Our aim was to explore the association between the level of maternal soluble HLA-G and the risk of malaria infection in their newborns. Here, 400 pregnant women and their children were actively followed-up during 24 months. The results show a significant association between the level of sHLA-G at the first antenatal visit and the time to first malaria infection during infancy adjusted to the risk of exposure to vector bites (aHR = 1.02, 95%CI [1.01–1.03], p = 0.014). The level of sHLA-G is a significant predictor of the occurrence of malaria infection during infancy consistent with the hypothesis that mother sHLA-G could be a biomarker of malaria susceptibility in children.

## Introduction

Children born to mother with placental malaria (PM) seem to have a shorter delay of occurrence of the first malaria infection^1–4^. This phenomenon is referred as immune tolerance (IT) that may be due to modifications of the newborn immune system development involving at least some cytokines production in cord blood^5^. These modifications may induce differential immune responses involving IL10 and Interferon-γ during infancy^6^. A similar phenomenon was described in cases of filariasis infection during pregnancy^7,8^. It has been shown that children born to mothers with PM were also at an increased risk for non-malarial fever^9^. Moreover, a recent result shows that children born to mothers with PM tend to have an increased risk for the first malaria attack but not for subsequent ones^10^. These observations highlight that it is a very complex phenomenon that could imply immunity to a broader sense, not specific to malaria infection and not only related to PM infection.

Human leucocyte antigen-G (HLA-G) is an immune-modulatory molecule that plays a crucial role in materno-foetal tolerance during pregnancy^11,12^ by interacting with immune cells of both innate and adaptive responses^13,14^. HLA-G is a non-classical HLA class I antigen, which differs from classical class I molecules in its restricted tissue distribution, diversity of protein isoforms and limited polymorphism^15,16^. The soluble isoforms detected in the plasma are shed HLA-G1 (HLA-G1s) and HLA-G5^11^. Apart from pregnancy, HLA-G has been described associated with chronic viral infections, cancer and *in vitro* fertilization success^17–22^. During viruses’ infections, HLA-G can be over-expressed by infected cells to create a tolerogenic environment helping the pathogen to escape immune system^23^. The same phenomenon has been described in several types of cancers^24^.

The association between HLA-G and the risk of malaria has been shown recently by our team at genetic^25,26^ and protein levels^27,28^. These last studies have been performed in different populations and geographic areas (Senegal in 2003 and Benin in 2010–2011). It clearly appears that the level of sHLA-G in children during the first year of life is strongly correlated with their own risk of malaria infection during the weeks following the measurement^28^. The sHLA-G level of a child was also associated with the sHLA-G level of his mother not only at delivery^28^ but also during the overall pregnancy^29^. Moreover, mothers with PM have a higher probability of giving birth to a child with a high level of sHLA-G during the first 2 years of life, independently of the mothers’ sHLA-G level^29^. Altogether, these results are consistent with both the fact that the risk of malaria infection during infancy is associated with the levels of sHLA-G in children and with the potential involvement of HLA-G in the immune tolerance phenomenon described during PM.

However, the direct association between the mothers’ levels of soluble HLA-G and the risk of malaria for their newborns remained unexplored. Here, our aim was to study the association between the mothers’ sHLA-G levels throughout the pregnancy and the risk of malaria during her newborn’s first 24 months of life.

## Results

### Descriptive results

Women’s mean age was 25.9 years (95% confidence interval [25.4–26.5]) and 15.7% were primigravid (Table 1). During the follow-up 16% of the mothers were infected by *P. falciparum* at first ANV (antenatal visit) and 4.9% at the second. Only two women were infected twice. The prevalence of PM was 10.8%. The mean level of soluble HLA-G at ANV1, ANV2 and at delivery are respectively 10.1 ng/ml (SD = 13.6), 10.6 ng/ml (SD = 14.0) and 17.3 ng/ml (SD = 34.6). Using multivariate linear regression, the level of sHLA-G did not differ significantly according to placental infection (p = 0.07, n = 370) or to peripheral infection at ANV1 (p = 0.67, n = 379), ANV2 (p = 0.71, n = 364) and at delivery (p = 0.26, n = 370).

**Table 1:** Characteristics of the Study Population at Inclusion.

Groups	Covariates	Characteristics
Mothers (*n* = 400)	Age (years)	25.9 SD = 5.4
	Gravidity	
	Primigravid	15.75%
	Multigravid	84.25%
	Placental malaria	10.8%
	Peripheral malaria	
	ANV1^(a)^	16.0%
	ANV2	4.9%
	Delivery	15.9%
	Ethnicity	
	Aïzo	69.50%
	Fon	20.75%
	Others	9.75%
	Married	97.8%
	ITP group^(b)^	
	SP	34.5%
	MQFD	35.5%
	MQSD	30.0%
	Health center	
	Sékou	76.0%
	Atogon	24.0%
Infants (*n* = 400)	Birth weight (grams)	3033.9 SD = 420.4
	Low birth weight	9.0%
	Gender	
	Female	53.0%
	Male	47.0%

^(a)^Antenatal visit.

^(b)^Two drugs were used for IPTp according to the protocol of the MIPPAD study: sulfadoxine-pyrimethamine (SP, 1500/75 mg) and mefloquine (MQ: 15 mg/kg), which is given once as a full dose (MQFD) or split over 2 days (MQSD).

In children, mean birth weight was 3034 g (95%CI, [2992.5–3075.4]), 9.0% of them had a LBW, and 14 (3.5%) were preterm.

During the study, 284 (71.0%) children developed at least one malaria episode (symptomatic or not): 50% of infected children developed two infections or more. Most of these infections were symptomatic (76.5%). There was no congenital infection.

At 12 months, 324 children had been followed-up and after 24 months, 189 infants had moved out of the area or were lost to follow-up. They were considered as censored observations. Finally all 400 infants were included in the survival analysis. The median duration of follow-up was 12 months.

### Cox model

All the variables respected the proportional hazards assumption (Table 2).

**Table 2:** Risk Factors of First Malaria in the First 2 Years of Life in Infants: Univariate and Multivariate Cox Analysis.

Covariates	Unadjusted HR	95% CI	*p*	Adjusted HR	95% CI	*p*
**Gender**						
Male	ref					
Female	0.85	0.7–1.1	0.16			
**Low birth weight**						
No	ref					
Yes	0.8	0.5–1.3	0.33			
**Environmental risk** ^(*)^	**1.3**	**1.1–1.5**	**<0.001**	**1.3**	**1.11–1.47**	**0.001**
**Maternal age**						
≤25 years	ref					
>25 years	1.001	0.99–1.01	0.87			
**Gravidity**						
Primigravid	ref					
Multigravid	0.99	0.7–1.4	0.95			
**Ethnic groups**						
Fon	ref					
Aïzo + others	**0.7**	**0.6–0.9**	**0.03**			
**IPT** ^(a)^						
SP	ref					
MQFD	1.2	0.9–1.6				
MQSD	0.9	0.7–1.2	0.07			
**Placental malaria**						
No	ref					
Yes	1.1	0.7–1.6	0.61			
**Peripheral infection at ANV1** ^(b,*)^	0.9	0.7–1.3	0.63			
**Peripheral infection at ANV2** ^(c,*)^	1.5	0.9–2.5	0.11			
**Peripheral infection at delivery** ^(*)^	1.13	0.8–1.5	0.43			
**sHLA-G at ANV1** ^(*)^	**1.01**	**1.00–1.02**	**0.05**	**1.02**	**1.01–1.032**	**0.014**
**sHLA-G at ANV2** ^(*)^	1.00	0.99–1.01	0.63			
**sHLA-G at delivery** ^(*)^	0.99	0.99–1.00	0.35			

^(*)^Continuous variable.

^**(a)**^Intermittent preventive treatment.

^**(b)**^Antenatal visit.

During univariate analysis, high environmental exposure was strongly associated with an increased risk of first infections (p < 10^−3^). Neither placental malaria nor the number of peripheral maternal infections during pregnancy was associated with the risk of first malaria. Infants from the “not Fon” ethnic group had a lower risk of infection (HR = 0.7, 95%CI [0.6–0.9], p = 0.03). The level of sHLA-G (used as a quantitative variable) at ANV1 was associated with the delay of first malaria infection (HR = 1.2, 95%CI [0.9–1.5], p = 0.05). At ANV2 and at delivery the association was not significant (p = 0.63 and p = 0.36 respectively). Finally, gender, environmental exposure, ethnic group and all variables concerning maternal HLA-G levels and malaria infections were included.

**Figure 1:**
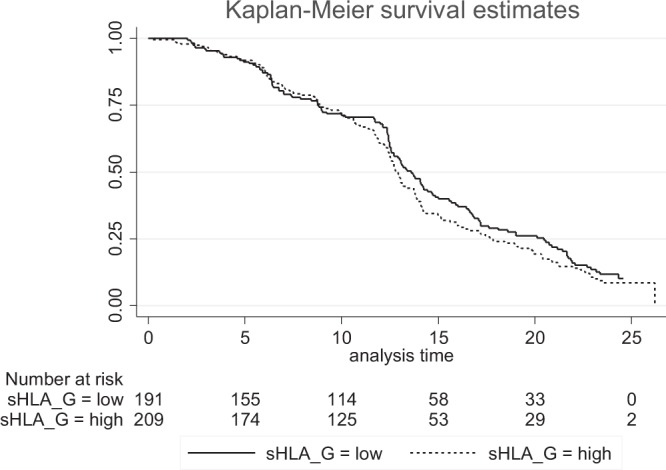
Kaplan-Meier survival curves in infants during the first 24 months of life according to the levels of sHLA-G in their mothers at the beginning of pregnancy. Plain line: low sHLA-G level (lower than the median value); dotted line: high sHLA-G level (higher than the median value).

During the multivariate analysis (Table 2), both environmental exposure to malaria (aHR = 1.3, 95%CI [1.11–1.48], p < 10^−3^) and s-HLAG at ANV1, used as quantitative variable, (aHR = 1.02, 95%CI [1.01–1.03], p = 0.01) remained significantly associated with the risk of first infection. This last result corresponds to the increase of risk of malaria infection when sHLA-G rises by 1 ng/mL. Using sHLA-G as a two-class variable (higher/lower that the median), this association was consistent with a 60% increased risk of presenting a first malaria infection for an infant born to a mother with high level of sHLA-G (aHR = 1.6, 95%CI [1.01–2.43, p = 0.04]) (not shown). The Kaplan-Meier curve (Fig. 1) shows that after 10 months of age, a high level of sHLA-G at the beginning of pregnancy was associated with an increased risk of a first malaria infection (Logrank test, p = 0.06).

### Predictive power

**Figure 2:**
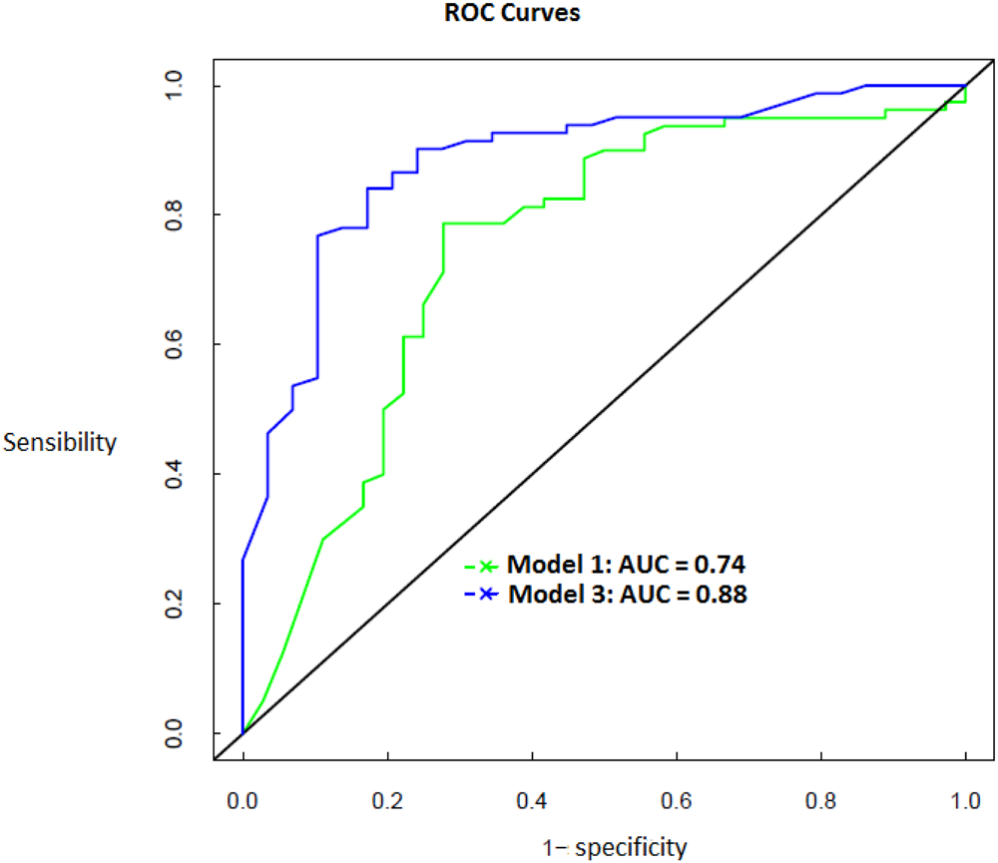
ROC curves of the reference model (logistic regression with all covariates except sHLA-G, model 1) and the best model (random forest with all covariates including sHLA-G (binary), (model 3). The best model (model 3) AUC is significantly higher than the reference model, showing that including the sHLA-G variable in the model increases the predictive power of the risk of a malaria infection occurring during the 2 first years of the babies.

The introduction of the level of sHLA-G of the mother at the first ANV in the predictive models increased significantly the predictive power of the risk of a malaria infection during the 24 first months of life. Indeed, the predictive models showed that significantly higher areas under the curves (AUC) were obtained in presence of sHLA-G in the model performed by both logistic regression and random forest (model 2 and model 3) compared to a model without sHLA-G (model 1). The best predictive power has been reached when sHLA-G was considered as a binary variable (lower or higher of the median value) by the 2 methods, and the highest AUC was obtained by the random forest method (model 3) (Fig. 2). The AUC were 0.74, 0.84 and 0.88 for model 1, model 2 and model 3, respectively, and Fig. 2 shows the ROC curves of the best model (model 3) vs. the reference model (model 1), with a significantly higher AUC of model 3 compared to the AUC of model 1 (p = 0.01, one-sided paired test). The conclusions were similar for all the stratified partitions performed at random, showing a good stability of the results.

## Discussion

The results are consistent with an association between the mothers’ level of soluble HLA-G at the first ANV and the time to first malaria infection during infancy. This association persists after adjustment on environmental risk of exposure. Moreover, it has been shown through machine learning predictive models that the level of sHLA-G at the first antenatal visit is a significant predictor of the risk of malaria infection during their first 2 years of life.

In Africa, differences in transmission levels exist at the very local level. Key determinants of local transmission intensity include vector profile, ecology and seasonality. Therefore, localized variations ought to be taken into account when considering the risk of infection in a population and the determinants of individual variability (i.e. behavior, physiology and genetics). In the present study, substantial variations in malaria vector density have been observed at the level of both village and house, even between houses which are close together. This variability could be explained not only by conventional climatic factors (rainfall, season) but also certain environmental factors, i.e. a watercourse nearby, and vegetation index and soil type in the immediate surroundings. The environmental risk of exposure has been taken into account very precisely by means of a predictive model that took into account both individual and environmental factors above-mentioned^30^. Interestingly, using this model it has been previously shown that the use of adequate prediction of this risk allows studying precisely the potential effect of placental infection in cohort studies^31^.The approach used in the present work is consistent with a suited management of the environmental risk of infection in this specific context of studying the effect of placental malaria and of other factors on the time to first malaria infection.

Several studies have concluded in the existence of a higher susceptibility to malaria for children born to mothers with PM^1–3^. The hypothesis proposed to explain this susceptibility is the existence of an immune tolerance status, although there is no clear and unequivocal explanation of the mechanism involved. The cord blood immune response of children born to mothers with PM is characterized by inducible parasite antigen-specific IL-10-producing regulatory T-cells that can inhibit Th1-type T cell response^32^. Interestingly, the same immune phenomenon involving an increased level of IL-10 in cord blood has been described during pregnancy geohelminth infections^33^. Moreover, it has been shown that children born to mothers with PM are also more susceptible to non-malaria fever^9^ strengthening the hypothesis that immune tolerance could surpass a specific parasite, and that PM could be a biomarker of a more generalized immunosuppressive phenomenon.

Human leukocyte antigen G can inhibit a broad array of immune cells and is strongly involved in fetal maternal tolerance during pregnancy^34^. A high level of expression of HLA-G, described as an immune checkpoint molecule^35^, is also reported when cancer, viral or parasitic infections occur^36,37^, favoring escape from immune control. Indeed, HLA-G interacts with immunoglobulin-like transcript 2 (ILT2) which is expressed by T and B lymphocytes, natural killer (NK) cells, monocytes/macrophages and dendritic cells, and with ILT4 expressed by monocytes, macrophages, neutrophils and dendritic cells)^38,39^. Binding of HLA-G proteins to their inhibitory receptors can affect the function of immune cell populations modulating crucial steps in immunity^40^ and inducing the differentiation of CD4^+^ and CD8^+^ T cells into various subsets of regulatory cells that secrete IL-10 and TGF-β^41,42^.

The origin of sHLA-G in the mother compartment is not clearly identified. During pregnancy, the source of plasmatic sHLA-G in mothers’ peripheral blood may come from the mother herself and/or from the fetal trophoblast cells. A child, a mother or both genetically committed to produce high levels of HLA-G could be more susceptible to infections. Therefore, the final sHLA-G production may be a combination of fetal–maternal HLA-G genotypes together with the micro-environmental factors that may modulate HLA-G expression^43^. In this context, PM may influence both mother and fetus HLA-G production inducing the immunological consequences listed above.

The increased malaria susceptibility of infants born of mothers with a high level of sHLA-G at the first ANV could be explained by several nonexclusive mechanisms. Firstly, genetic variants of the infant involved in the regulation of sHLA-G expression influence the level of sHLA-G in the mother during pregnancy (via trophoblast cells) and in the children during infancy^43^. A high level of sHLA-G represents a tolerogenic environment from the infant’ immune system and increases the risk of infection^27,28^. The correlation between the level of mothers’ sHLA-G during pregnancy and in the infant during the first 2 years of life^28^ could be explained by these genetic factors. Secondly, high levels of sHLA-G observed at ANV1 are induced by the presence of pathogen (not necessarily malaria) escaping the mothers’ immune system and also represents a tolerogenic environment from the infant’s immune system. Infants exposed to infection during pregnancy, combined with a strong immune system regulation on the part of the mother, may develop an attenuated immune response towards pathogens. This state of unresponsiveness or weakness of the infant’s immune system does not seem pathogen-specific but a more generalized phenomenon^9^.

The absence of associations between the time to the first malaria infection during infancy and the mothers’ levels of sHLA-G at ANV2 and delivery could be explained by the medical care provided to the mother, including the two doses of IPTp against malaria at each antenatal visit. They were also encouraged to attend the clinic at any time, whenever they had any health complaint. These different treatments received during pregnancy may also have modulated HLA-G expression due to the reduction of exposure to infectious diseases. However, independently of the possible effect of IPT or of other treatment received during the follow-up, it is essential to underline the importance of the beginning of pregnancy *per se*. Indeed there is accumulating evidence that, although the pathophysiological mechanisms of early infections are not completely understood yet, their consequences are serious in terms of maternal anemia and low birth weight^44^. Moreover, it has been shown that even submicroscopic *P. falciparum* infections can have consequences in terms of low birth weight, prematurity and maternal anemia^45^. To our knowledge the consequences of malaria infection of the mothers during early pregnancy on the susceptibility of their newborns to infection during infancy have been poorly explored and the results are not convincing^46^. Our results could be indirectly consistent with the potential existence of such an association and by the involvement of HLA-G in this susceptibility.

It has been shown that the level of HLA-G at ANV1 has a significant predictive power of the occurrence of malaria infection during the first 2 years of life. This finding is promising in that it constitutes a strong argument for the idea that maternal HLA-G level could be a biomarker for malaria susceptibility in children. This question must, however, still be developed by complementary studies.

Some limitations of the study exist. Due to the constraints imposed by the clinical trial, the follow-up was different before and after the first 12 months. Therefore, there is a possibility that some infections could have been missed during the 1st year because of less extensive monitoring. Nevertheless, this possibility should in particular concern asymptomatic infections since mothers received the instruction to contact health centers if any health problems arose. However under-detection of malaria events is unlikely to be related to the maternal HLA-G level and this should not constitute a major and particularly specific bias in the results. Furthermore, this could explain why the effect of sHLA-G level seems more detectable after 10 months of life. Nevertheless, the same pattern of results was obtained using symptomatic infections alone (p = 0.03).

HLA-G genetic polymorphism could influence HLA-G expression^47,48^ and consequently modulate the risk of malaria infection^49^. We have studied the role played by genetic polymorphism of *HLA-G* 3′UTR regulatory region. No association was observed between *HLA-G* 3′UTR variants and the level of sHLA-G expression (data not showed). However we cannot exclude that genetic variants present in *HLA-G* 3′UTR could modulate HLA-G concentration because the absence of association observed is most likely due to insufficient number of individuals. Moreover, genetic variants in others regulatory regions could also affect HLA-G expression^50,51^. Studies combining *in vitro* functional assays and larger cohorts are needed to better understand the role played by genetic on HLA-G expression.

The potential advantage of soluble HLA-G as a biomarker has already been proposed in cancer, chronic viral infections and *in vitro* fertilization^20–22^. Our results are consistent with the potential value of sHLA-G from a public health point of view to identify pregnancies that could result in particularly frail newborns. However, we believe it is too early to make some recommendation for the follow-up of women and newborn, and we need to confirm these results in another population including a higher number of pregnant women.

## Material and Methods

### Study site and population

The present prospective cohort study took part in the framework of the MiPPAD clinical trial (http://clinicaltrials.gov/ct2/show/NCT00811421). The first 400 infants to be delivered were enrolled from January 2010 to June 2011, and followed up for 24 months. HIV-positive, twin pregnancies, stillbirth or fetal abnormalities were excluded. Women were included before the end of 28 gestational weeks (GW) and two doses of Intermittent Preventive Treatment for pregnancy (IPTp) were administered at antenatal visits (ANVs)^52^. In our analyses ANV1 and ANV2 referred respectively to the ANVs during which the first and the second IPTp doses were given, in compliance with the MiPPAD clinical trial protocol.

### Study procedures

At inclusion, socio-demographic and socio-economic characteristics, reproductive and medical histories were collected. Women were examined and a questionnaire completed. Between ANVs, women had to attend the health centre for all health complaints.

Blood was sampled (before IPTp administrations at ANV1 and ANV2 and before delivery) for plasma sHLA-G measurement and malaria diagnosis. After delivery, a placental blood smear was used to assess placental malaria, defined as the presence of asexual *Plasmodium falciparum* parasites in the blood smear.

Newborns were followed until 24 months. Due to the MiPPAD clinical trial constraints, the follow-up was different before and after 12 months. During the first year of life, at 6, 9 and 12 months children were clinically examined. In case of axillary temperature greater than or equal to 37.5 °C (or a history of fever in the preceding 24 h) a rapid diagnosis test (RDT) and a thick blood smear (TBS) were performed. After 12 months of age, children were visited at home twice a month and the temperature was systematically checked. During the 24-month period, mothers were invited to visit health centers if there was any health problem. During home visits or at the health center, in case of fever or history of fever in the preceding 24 h, a RDT was performed. Monthly between 12 and 24 months, a systematic TBS to detect asymptomatic malaria was also performed. A symptomatic malaria attack was defined as the presence of fever (or a history of fever) and a positive RDT and/or a positive TBS. Malaria attacks were treated with an artemisinin-based combination (arthemeter and lumefantrine), as recommended by the Beninese National Malaria Control Program. An asymptomatic infection was defined as a positive monthly systematic TBS with no fever or history of fever. Overall, at birth (cord blood), 6, 9, 12, 18 and 24 months, blood was collected to perform the same tests evaluated in mothers. All the medications prescribed were free of charge.

### Soluble HLA-G quantification

Soluble HLA-G was quantified using the MEM-G/9 antibody (Exbio, Praha, Czech Republic), which recognizes the sHLA-G1, -G5 isoforms and the anti-human β2-microglobulin, as capture and detection antibodies, respectively^53^. All incubation steps were performed at room temperature, followed by four washes using washing buffer (H_2_O, PBS 1X, 0.1% Tween 20). The plates were incubated for 30 min with the substrate (Tetramethylbenzidine, Sigma Aldrich, St. Louis, MO, USA) and absorbance was measured at 450 nm after adding HCL (1 N). Total sHLA-G levels were determined from a five-point standard curve (12.5–200 ng/mL) using dilutions of calibrated HLA-G5 purified from M8-HLA-G5 cell line culture supernatant, and the results were expressed as ng/mL. The detection limit is ~1 ng/mL. A negative and positive control was included in each ELISA plate. The positive control was the supernatant from M8-HLA-G5 cell line culture (with the vector encoding the secreted protein HLA-G5 (M8-HLA-G5)) while the negative control was the supernatant from M8 cell line transfected with the empty vector (M8-pcDNA).). Both M8-HLA-G5 and M8-pcDNA cell lines were previously described^54^. The precise concentration of HLA-G5 present into the M8-HLA-G5 supernatant was defined using HLA-G5 purified protein^55^. The methodology to measure sHLA-G1 and -G5 molecules using ELISA has been previously validated the Wet Workshop for measurement of sHLA-G held in Essen, Germany and published^56^.

### Definition of variables

For survival analysis, time between birth and first malaria infection (symptomatic or not) was defined as primary outcome. After their first malaria infection, children were right-censored. The date of right censoring was the date of first malaria infection or the last available date of follow-up.

The following variables were used. Gender of the newborn; presence of LBW (birth weight <2500 g); age of the mother; gravidity (primi vs multigravid); ethnic group (Aizo, Fon, other); IPTp treatment group; placental malaria infection (presence/absence); number of peripheral malaria infections during pregnancy (before ANV1, between ANV1 and ANV2 and after ANV2); level of sHLA-G at ANV1, at ANV2 and at delivery. Levels of sHLA-G at each measurement were used as quantitative variable. The environmental risk of infection was assessed through quantification of exposure to vector bites. Mosquito catches were performed over two consecutive nights, monthly throughout the course of the study, to assess the density of malaria vectors. Using environmental (rainfall, type of soil, watercourse nearby, vegetation index) and behavioral data (number of inhabitants per room; ownership of a bed net or insect repellent), a time- and space-dependent environmental risk of exposure to malaria was assessed for each child by means of a predictive model^30^.

### Statistical analyses

#### Survival analyses

A Cox regression model was used to assess the effect of PM and of the mothers’ sHLA-G levels on the time of first infection, adjusted on the cofactors mentioned above. First, a univariate Cox analysis was performed to study the association between all covariates and the first malaria infection. Kaplan-Meier curves were obtained to present the probability of occurrence of malaria infection. Secondly, a multivariate analysis was performed to study the association between first malaria infection and PM or mothers’ HLA-G levels, adjusting for the covariates selected in the first step (with *p* < 0.20). Scaled Schoenfeld residuals were used for testing the proportional-hazards assumption.

Data were analyzed using the Stata v. 13 software (Stata Corporation, College Station, TX, USA).

#### Predictive analyses

Complementary, we built a predictive model, designed to evaluate the power of sHLA-G to predict the occurrence of infection during the 2 years follow-up (no infection vs at least one). We performed two machine-learning methods: logistic regression and random forest. For both methods, the same steps were followed. First, the sample was split into a train sample (70% of the data) and a test sample (30% of the data) randomly drawn with stratification according to the malaria infection status to guarantee the same proportion of infection in both samples. At a second step different predictive models were built (see details below) using the train sample and at the last step, the predictive power of each model was evaluated using the test sample through the area under ROC curves (AUC). All the covariates were introduced in the predictive models, which differed only according to the way the sHLA-G variable has been considered: (i) sHLA-G as a binary variable (2 classes according to the median, (ii) sHLA-G as a four classes variable (according to the quartiles) and (iii) sHLA-G as a continuous variable. Then, the best sHLA-G variable was selected (i.e. the sHLA-G variable giving the highest predictive power (highest AUC) was selected. At last, three models were compared by testing the difference between the areas under the ROC curves: logistic regression with all covariates except sHLA-G (model 1 considered as the reference model), logistic regression with all covariates including the best sHLA-G variable (model 2) and random forest with all covariates including the best sHLA-G variable (model 3). Finally, the stability of the results has been checked by repeating this last step on 4 other partitions of the data drawn at random (i.e. 5 pairs of train/test samples in all). These analyses were performed using R software, (MLR package).

### Ethics

The study protocol and informed consent were approved by the *Comité Consultatif de Déontologie et d'Éthique* (CCDE) of the *Institut de Recherche pour le Développement* (France) and by the *Ethics Committee of the Faculté des Sciences de la Santé de Cotonou* in Benin (N° 43/11/2010/CE/FSS/UAC). Written informed consent was signed and a copy was given to each participant with the possibility to withdraw at any time. If the woman could not read, an impartial witness was involved in the process. In addition to the assent of minors, informed consent was obtained from the parents or legal guardians. All the methods were carried out in accordance with the approved guidelines.

## Data Availability

All relevant data are available from the Open Science Framework database (https://osf.io/q2v57/).
